# *Lactobacillus delbrueckii* ssp. *bulgaricus* OLL1073R-1-derived exopolysaccharide suppresses murine norovirus replication by modulating innate immune response

**DOI:** 10.1371/journal.pone.0356886

**Published:** 2026-09-02

**Authors:** Shuyi Tang, Shoko Ishii, Reiko Takai-Todaka, Miho Ogawa, Reiko Watanabe, Hiroshi Kano, Toshihiro Sashihara, Kenichi Hojo, Kazuhiko Katayama, Kei Haga

**Affiliations:** 1 Food Microbiology and Function Research Laboratories, R&D Division, Meiji Co., Ltd., Nanakuni, Hachioji, Tokyo, Japan; 2 Laboratory of Viral Infection Control, Ōmura Satoshi Memorial Institute, Graduate School of Infection Control Sciences, Kitasato University, Shirokane, Minato-ku, Tokyo, Japan; Defense Threat Reduction Agency, UNITED STATES OF AMERICA

## Abstract

Norovirus is a major cause of foodborne acute gastroenteritis, yet effective strategies to prevent norovirus infection have not been established. Given that an exopolysaccharide (EPS) can be orally consumed as a dietary component, we evaluated its potential to suppress norovirus replication *in vitro*. Here, we tested the antiviral effect of EPS derived from *Lactobacillus delbrueckii* ssp. *bulgaricus* OLL1073R-1 (R-1 EPS) against murine norovirus (MNV) *in vitro* using the mouse macrophage-like cell line, RAW264.7. Post-infection treatment significantly reduced the viral load of MNV in culture supernatants. Microarray-based transcriptome analysis showed that R-1 EPS treatment modulated MNV-induced host transcriptional responses, promoting preferential engagement of interferon (IFN)-mediated antiviral and inflammatory pathways. Consistent with the microarray-based transcriptome analysis, expression analysis using ELISA and RT-qPCR indicated increased expression of IFN, IFN-stimulated genes, and proinflammatory cytokines in infected cells treated with R-1 EPS. The results suggest that R-1 EPS modulated host innate immune responses, such as type I IFN and proinflammatory cytokine responses, during MNV infection, which may contribute to antiviral activity. Our results provide mechanistic insights into the host-directed antiviral effect of microbial EPS.

## Introduction

Norovirus is a highly contagious viral pathogen that causes foodborne illnesses and acute gastroenteritis, especially in winter. The typical symptoms of norovirus infection include vomiting, abdominal cramps, and watery diarrhea [1,2]. In healthy individuals, the symptoms can occur within a short period of 12–48 h after exposure [3]. Most people fully recover from the symptoms within 2–3 days; however, some individuals, especially infants, older individuals, and individuals with weakened immune systems, are at greater risk for severe symptoms and complications [4]. Thus, norovirus poses a major public health risk; however, no effective medications are available for norovirus infection, and because of the diverse genetic types and technical challenges in culturing norovirus, an effective vaccine has not yet been developed. Therefore, we aimed to develop an effective strategy to prevent norovirus infection by focusing on the activation of host innate immunity. Antiviral approaches that enhance host innate immune responses may exhibit broad-spectrum antiviral activity against various viral pathogens when compared with strategies that directly target viral proteins [5,6]. Accordingly, such approaches represent a promising antiviral strategy for genetically-diverse viruses such as norovirus.

Many aspects of human norovirus (HuNoV) biology are poorly understood, largely because of the lack of a suitable cell culture system or small-animal model. Murine norovirus (MNV), which is closely-related genetically to HuNoV and can easily propagate in cell culture systems, has been used as a surrogate virus for HuNoV [7–10]. MNV and HuNoV are both non‑enveloped, positive‑sense, single‑stranded RNA viruses with a genome length of approximately 7.5 kb and contain three (HuNoV) or four (MNV) open reading frames (ORFs) [7,11]. While this varies according to host cell susceptibility, MNV and HuNoVs complete their replication cycles within 24 h [12,13]. Although recent advances have reported a human enteroid-based culture system for HuNoV [13], simple and convenient cell culture systems and small-animal models of HuNoV are lacking. In contrast, MNV can be efficiently propagated in a well-established culture system using the mouse macrophage-like cell line, RAW264.7 [8], which is experimentally straightforward and highly reproducible. This practical advantage has made MNV a widely-used and valuable model for investigating norovirus replication and virus–host interactions.

In both HuNoV and MNV, the P2 domain of the major capsid protein, VP1, is highly diverse, which is implicated in differences in host receptor recognition and antigenicity [12,14]. MNV infects host cells, such as macrophages and dendritic cells, utilizing the receptors, CD300If and CD300Id [15–17], whereas HuNoV primarily infects epithelial and B cells, with histo-blood group antigens (HBGAs) presumed to serve as their receptor [18–21]. These differences in the target cells and receptors influence the entry mechanism, cell tropism, and sensitivity of the virus to immune responses. Despite these differences, MNV offers practical advantages, including the availability of a robust cell culture system, and both MNV and HuNoV have been reported to show susceptibility to innate immune responses such as type I interferon (IFN) [22,23]. Therefore, MNV was selected as a suitable model for the initial stage of the current investigation.

Macrophages are key innate immune cells that detect viral invasion and stimulate adaptive immune responses. Macrophages detect pathogen-associated molecular patterns (PAMPs) using pattern recognition receptors (PRRs), such as Toll-like receptors (TLRs) and retinoic acid-inducible gene (RIG-I)-like receptors (RLRs). This recognition activates signaling pathways, involving nuclear factor-kappa B (NF-κB), interferon-regulatory factor (IRF), thereby resulting the production of type I interferons (IFNs) and cytokines [24]. Type I IFNs subsequently activate the Janus kinase (JAK)/signal transducer and activator of transcription (STAT) pathway, which induces the expression of interferon-stimulated genes (ISGs) that mediate antiviral responses and restrict viral replication [25,26]. These responses shift cells into an antiviral state and are essential for limiting viral replication. Thus, the use of macrophage-like cell lines for investigation aligns with the objective of the present study, which focused on innate immunity. Moreover, MNV enables efficient replication and spread by evading or suppressing the defense mechanisms of macrophages, a process that may be achieved in part through the inhibition of PRR-mediated signaling [24] and the induction of anti-inflammatory regulatory pathways [27].

Exopolysaccharides (EPS) are high-molecular-weight polysaccharides secreted by lactic acid bacteria during fermentation. Their chemical structures vary among bacterial strains and species due to difference in sugar composition, branching pattern, and chemical modification, resulting in diverse biological functions and their health-promoting effects [28]. EPS have been reported to exhibit various functions, including antibacterial and antioxidant activities, regulation of gut microbiota, immune modulation, regulation of lipid metabolism, and anti-tumor properties [29,30]. Notably, EPS are known to modulate immune functions [31,32]. Although the mechanisms underlying these effects differ among polysaccharides, reported effects include modulation of receptor signaling [33,34], regulation of cytokine production and immune cell functions [31,35], and direct interaction with pathogens [36,37]. In this study, we focused on EPS produced by *Lactobacillus delbrueckii* ssp. *bulgaricus* OLL1073R-1 (R-1 EPS). This strain was selected from 139 *L*. *bulgaricus* strains because of its high EPS-producing capacity and potent immunostimulatory activity, particularly its ability to induce IFN-γ production [38]. In addition, R-1 EPS has been shown to exhibit antiviral effects by activating innate immune responses, including protection against influenza virus infection [39,40]. It has also been reported that R-1 EPS induces type I IFN gene expression in a model using porcine intestinal epithelial cells and Poly(I:C) [41]. However, its effect on enteric viral replication has not yet been evaluated. Clinically relevant enteric viruses, including norovirus, are non-enveloped and structurally adapted to remain stable and infectious in the gastrointestinal environment. Although norovirus is highly stable under such conditions, its replication is strongly restricted by innate immune response, particularly IFN signaling [23]. Because R-1 EPS is orally ingested as a food component and is expected to function in the gastrointestinal tract, we investigated whether R-1 EPS can suppress norovirus replication through modulation of host innate immune response using a MNV infection model in mouse macrophages. Our results demonstrated that R-1 EPS suppresses MNV replication while enhancing the type I IFN response in MNV-infected RAW264.7 cells.

## Materials and methods

### Cells and viruses

HEK293T cells (American Type Culture Collection (ATCC), Manassas, VA, USA) were maintained in Dulbecco’s modified Eagle’s medium (DMEM; Fujifilm Wako Pure Chemical, Osaka, Japan) supplemented with heat-inactivated (at 56°C for 1 h) 10% fetal bovine serum (FBS; Biowest, Nuaillé, France), 100 U/mL penicillin, and 100 µg/mL streptomycin (Gibco, Waltham, MA, USA) at 37°C under 5% CO_2_. A cell line derived from mouse leukemic monocyte macrophages, RAW264.7 (ATCC), was grown in DMEM (Fujifilm Wako Pure Chemical) supplemented with heat-inactivated (at 60°C for 1 h) 10% FBS (Biowest), 100 U/mL penicillin, 100 µg/mL streptomycin (Gibco), and 5 mM HCl (Fujifilm Wako Pure Chemical) at 37°C under 5% CO_2_.

MNV-S7 strain was produced using a plasmid-based reverse-genetic system in HEK293T cells and then propagated in RAW264.7 cells [42].

### Purification of exopolysaccharides

R-1 EPS was prepared following a previously described method [39]. Briefly, skim milk fermented with *L. delbrueckii* ssp. *bulgaricus* OLL1073R-1 was mixed with trichloroacetic acid to remove proteins, and ethanol precipitation was performed. Crude R-1 EPS was dialyzed (molecular weight cut-off: 6,000–8,000 Da) and incubated with RNase, DNase, and proteinase K. After ethanol precipitation and dialysis, R-1 EPS was obtained by freeze-drying. For use in the experiments, the freeze-dried R-1 EPS was suspended in an appropriate medium for each experiment and dissolved completely by incubating at 4°C, overnight.

### Viral growth inhibition assay

RAW264.7 cells were seeded in a 12-well tissue culture plate and incubated for 24 h. The cells were then infected with MNV-S7 at a multiplicity of infection (MOI) of 1 and incubated for 2 h at 4°C. After incubation, the cells were washed with phosphate buffered saline once. The infected cells were incubated in growth medium (untreated control) or medium containing 200 µg/mL R-1 EPS for 36 h. The cell culture supernatant was collected every 3 h for analysis.

### Quantification of MNV load

Genomic RNA copies of MNV in the cell culture supernatant were quantified using a quantitative reverse transcription-polymerase chain reaction (RT-qPCR) assay, as previously reported [43]. Briefly, MNV RNA was extracted from 140 µL of cell culture supernatant using the QIAamp Viral RNA Mini Kit (Qiagen, Hilden, Germany). The extracted RNA was incubated with RQ1 RNase-free DNase (Promega, Madison, WI, USA) for DNA degradation, and complementary DNA (cDNA) was generated via reverse transcription using the High-Capacity cDNA Reverse Transcription Kit (Applied Biosystems, Waltham, MA, USA). RT-qPCR was performed using the QuantiTect Probe PCR kit (QIAGEN) on the ABI 7300 Real-Time PCR System (Applied Biosystems). The following MNV-specific primers and probe were used: forward primer 5′- CCGCAGGAACGCTCAGCAG-3′, reverse primer 5′- GGYTGAATGGGGACGGCCTG-3′, and probe FAM-5′- ATGAGTGATGGCGCA-3′-MGB NFQ. A plasmid containing the full-length MNV-S7 cDNA was serially diluted to obtain a standard curve.

### Microarray analysis

MNV-infected RAW264.7 cells were collected at 9 h post-infection (hpi), and total RNA was extracted and purified using the RNeasy Mini Kit (Qiagen). RNA was extracted from triplicate samples and combined into one sample. Microarray analysis was performed using a TORAY 3D-gene oligo chip (TORAY, Tokyo, Japan) for mRNA with a dual-color system, according to the manufacturer’s instructions. RNA isolated from non-infected cells was co-hybridized on the same chip and used as a reference to normalize gene expression levels in the R-1 EPS-untreated and -treated infected groups. Raw data are included in the supporting information (S1 data).

For differential expression analysis, differentially expressed genes (DEGs) were identified by comparing gene expression levels between MNV-infected (MNV) or MNV-infected R-1 EPS-treated (MNV + R-1 EPS) cells and uninfected cells (Mock), and genes with a log_2_ fold change [MNV/Mock or MNV + R-1 EPS/Mock] ≥ 1 or ≤ −1 were defined as DEGs. Enrichment analyses of upstream transcription factor, Gene Ontology (GO), and pathway were performed using Enrichr (https://maayanlab.cloud/Enrichr/) [44–46]. Visualization was performed using R (v4.3.1) [47] and the R package ggplot2 [48].

### Quantification of cytokines

The concentrations of IFN-α, IFN-β and TNF-α in culture supernatant were determined using the VeriKine Mouse Interferon alpha ELISA Kit (PBL Assay Science, Piscataway, NJ, USA), Quantikine ELISA Mouse IFN-β Kit (R&D Systems, Minneapolis, MN, USA), and Mouse TNF-alpha ELISA Kit (Proteintech, Rosemont, IL, USA), respectively, according to the manufacturers’ protocol.

### Gene expression analysis of ISGs and factors involved in type I IFN signal transduction

MNV-infected RAW264.7 cells were collected every 3 h until 18 hpi, and total RNA was extracted and purified using RNeasy Mini Kit (Qiagen) for gene expression analysis. Total RNA was extracted using the RNeasy mini kit (Qiagen) and cDNA was prepared using PrimeScript™ RT Master Mix (Takara Bio, Ohtsu, Japan) according to the manufacturer’s protocol. RT-qPCR was performed using TB Green^®^ Premix Ex Taq™ II (Takara Bio) with the Quantstudio^®^ 3 system (Thermo Fisher Scientific, Waltham, MA, USA). The specific primers used in the expression analysis are shown in Table 1. The gene expression was quantified using the 2^−ΔΔCT^ method and normalized to the level of a constitutively expressed housekeeping gene encoding hypoxanthine phosphoribosyltransferase (*HPRT*).

**Table 1:** Specific primers used in the expression analysis of interferon-stimulated genes (ISGs) and factors involved in type I IFN signal transduction.

Target gene	Forward/Reverse	Sequence (5’-3’)
***HPRT***	Forward	TTGTTGTTGGATATGCCCTTGACTA
	Reverse	AGGCAGATGGCCACAGGACTA
***ISG15***	Forward	CAATGGCCTGGGACCTAAA
	Reverse	CTTCTTCAGTTCTGACACCGTCAT
***Viperin***	Forward	TGCTGGCTGAGAATAGCATTAGG
	Reverse	GCTGAGTGCTGTTCCCATCT
***OAS***	Forward	GAGGCGGTTGGCTGAAGAGG
	Reverse	GAGGAAGGCTGGCTGTGATTGG
***Mx1***	Forward	ACAAGCACAGGAAACCGTATCAG
	Reverse	AGGCAGTTTGGACCATCTTAGTG
***MDA5***	Forward	AGTGTCAGCTGCTTCGATGA
	Reverse	ATTTGGTAAGGCCTGAGCTG
***RIG-I***	Forward	GCAGACATGGGATGAAATGA
	Reverse	TCTTGCACTTTCCACACAGC
***IRF1***	Forward	CCCACAGAAGAGCATAGCAC
	Reverse	AGCAGTTCTTTGGGAATAGG
***IRF7***	Forward	GTCTCGGCTTGTGCTTGTCT
	Reverse	CCAGGTCCATGAGGAAGTGT

### Statistical analysis

All statistical analyses were performed on GraphPad Prism version 10.0 for Windows (GraphPad Software). The data were analyzed using two-way ANOVA, followed by Sidak’s post-hoc test vs. the corresponding control. Statistical significance was set at *P*  <  0.05. Biological replicates were defined as independent experiments that were conducted using separately-prepared cell cultures. Technical replicates represent repeated measurements of the same biological samples. Statistical comparisons were conducted on technical triplicates within representative experiments. Representative data from multiple biological replicates are shown. The numbers of biological replicates for each experiment are specified in the corresponding figure legends.

## Results

**Fig 1:**
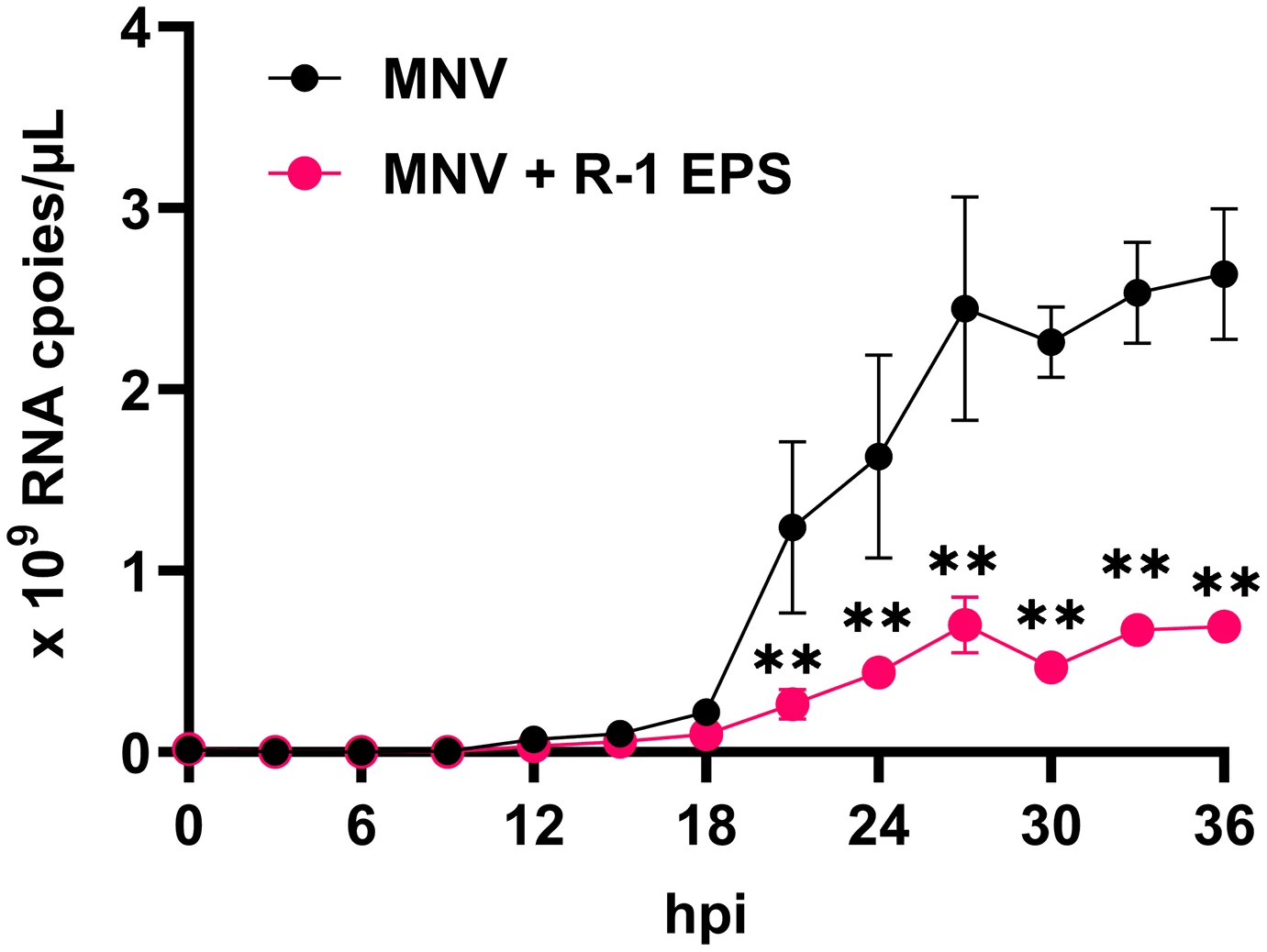
R-1 EPS suppressed MNV production in RAW264.7 cell culture supernatant. RAW264.7 cells were infected with murine norovirus (MNV-S7 strain) and subsequently treated with 200 µg/mL R-1 EPS (MNV + R-1 EPS). Cells infected and cultured without R-1 EPS were used as untreated control (MNV). The culture supernatant was collected every 3 h and viral RNA copy numbers were quantified using RT-qPCR. Black circles represent the MNV and magenta circles represent the MNV + R-1 EPS group. Data are presented as geometric mean ± standard deviation (SD) of triplicate wells per group. Experiments were performed with two independent biological replicates, and representative data are shown. **: *P* < 0.01 (two-way ANOVA with Sidak’s post-hoc test vs. the corresponding control).

To verify the inhibitory effect of R-1 EPS on MNV infection, mouse macrophage-like cells (RAW264.7) were infected with MNV and subsequently cultured in the presence of 200 µg/mL of R-1 EPS. Previous research has shown that stimulating human peripheral blood mononuclear cells (PBMCs) with R-1 EPS at concentrations of 100 μg/mL or higher significantly induces IFN-γ production, with levels reaching a plateau above this concentration [49]. Furthermore, in our previous studies, we have reported that stimulating human PBMCs with R-1 EPS at 100 µg/mL induces the production of IFN-β [50]. Considering the possibility that the immune response may be weaker in RAW264.7 cells, which are cultured cell, compared to human PBMCs, the concentration of R-1 EPS was set to a higher dose of 200 μg/mL. We confirmed that the treatment of RAW264.7 cells with 200 µg/mL of R-1 EPS did not show any cytotoxicity (S1 Fig). The cell culture supernatant was collected every 3 h and viral RNA copy numbers in the cell culture supernatant was quantified using RT-qPCR. A significant reduction in MNV RNA copy number was observed in cell culture supernatant treated with R-1 EPS (MNV + R-1 EPS) from 21 to 36 hpi compared to untreated RAW264.7 cell culture supernatant (MNV) (Fig 1), with the maximum inhibition rate of 79.3% observed at 30 hpi (S1 Table). This result suggests that R-1 EPS suppressed MNV replication.

Marked inhibition of MNV replication by R-1 EPS was evident at 21 h post-infection and thereafter. At this time point, viral RNA levels were markedly increased compared to those at earlier time points (≤18 h), a phase that is generally associated with the release of progeny virions and their subsequent spread to neighboring cells. Therefore, the reduction in the viral load observed at this stage raised the possibility that R-1 EPS may interact directly with newly-released virions and exert virucidal activity. To determine whether R-1 EPS targeted viral particles directly, MNV was pre-incubated with R-1 EPS prior to infection in the absence of host cells. This treatment did not alter viral infectivity (S2 Fig), suggesting that the antiviral effect of R-1 EPS was unlikely to be mediated by direct virion inactivation and was instead consistent with a host-directed mechanism.

Microarray analysis was conducted to elucidate the mechanism by which R-1 EPS suppresses MNV replication. The analysis was conducted using a dual-color microarray to simultaneously compare the expression levels in non-infected (Mock), untreated infected (MNV), and R-1 EPS-treated infected cells (MNV + R-1 EPS). A 9-hpi sample was used for microarray analysis. Intracellular infectious MNV progeny particles become detectable at approximately 12-h post-infection, suggesting that the eclipse phase extends close to this time point [12]. In our experimental system, viral levels were largely unchanged at 3 and 6 hpi, whereas a slight increase in viral RNA levels (approximately 60-fold) without R-1 EPS treatment was observed at 9 hpi, as shown using RT-qPCR analysis (Fig 1, S1 Table). This indicates that 9 hpi corresponds to a transitional stage when viral replication becomes detectable but precedes robust virion accumulation, and we selected this time point to capture early host transcriptional responses associated with the onset of viral replication, while minimizing secondary effects arising from extensive viral propagation and cytopathic effects.

**Fig 2:**
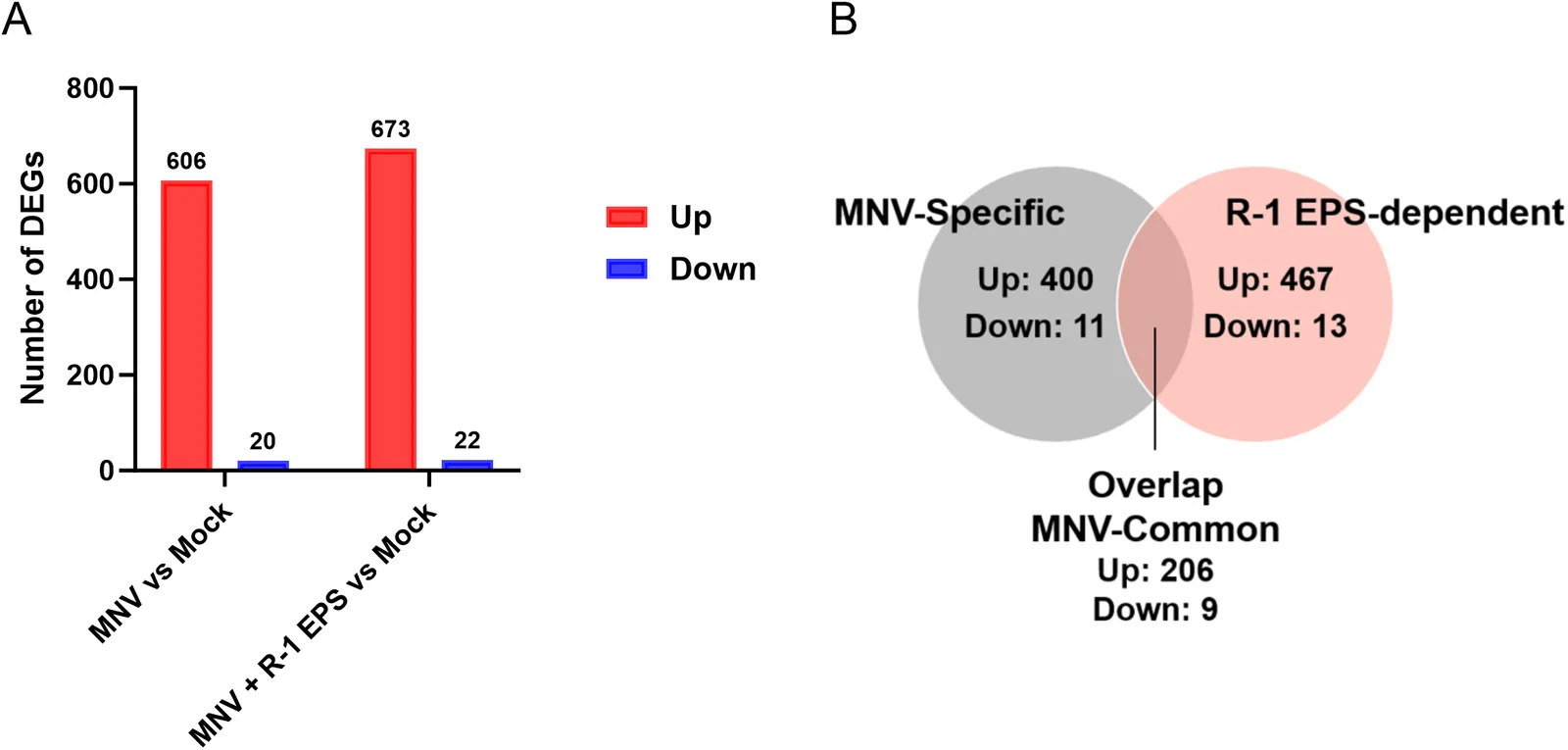
Differential gene expression analysis in MNV-infected cells without or with R-1 EPS. RAW264.7 cells were infected with MNV-S7 and subsequently treated without (MNV) or with 200 µg/mL R-1 EPS (MNV + R-1 EPS). Non-infected cells were used as a control (Mock). The cellular RNA was collected at 9 h post-infection (hpi) for microarray analysis. (A) Number of differentially expressed genes (DEGs) identified in the MNV vs Mock and MNV + R-1 EPS vs Mock comparisons. Upregulated and downregulated genes (log_2_ fold change ≥ 1 or ≤ −1) are shown as bar graphs. (B) Venn diagrams illustrating the overlap and unique DEGs between the MNV and the MNV + R-1 EPS groups. The overlapping regions represent genes commonly-regulated by MNV infection irrespective of R-1 EPS treatment (MNV-common), whereas non-overlapping regions indicate genes uniquely-regulated in the MNV (MNV-specific) and the MNV + R-1 EPS groups (R-1 EPS-dependent).

Compared to Mock, genes with expression levels at least two-fold higher or lower (log_2_ fold change [MNV/Mock or MNV + R-1 EPS/Mock] ≥ 1 or ≤ −1) were identified as differentially expressed genes (DEGs). A total of 606 and 673 DEGs were upregulated in the MNV and MNV + R-1 EPS groups, respectively, whereas genes 20 and 22 were downregulated in the MNV and MNV + R-1 EPS groups, respectively (Fig 2A).

To distinguish genes commonly-regulated by MNV infection independent of R-1 EPS treatment from those uniquely-regulated by R-1 EPS during viral infection, DEGs that overlapped between the MNV and MNV + R-1 EPS groups, as well as those uniquely identified in each group were investigated. A total of 206 genes were upregulated in both MNV and MNV + R-1 EPS groups (hereafter referred to as MNV-common DEGs). In contrast, 400 genes were specifically upregulated in the MNV group (MNV-specific DEGs), whereas 467 genes were uniquely upregulated in the MNV + R-1 EPS group (R-1 EPS-dependent DEGs). Similarly, nine genes were commonly downregulated in MNV-common DEGs, whereas 11 and 13 genes were downregulated exclusively in MNV-specific and R-1 EPS-dependent DEGs, respectively (Fig 2B). Because the vast majority of DEGs exhibited increased expression, subsequent analyses focused exclusively on the upregulated genes.

**Fig 3:**
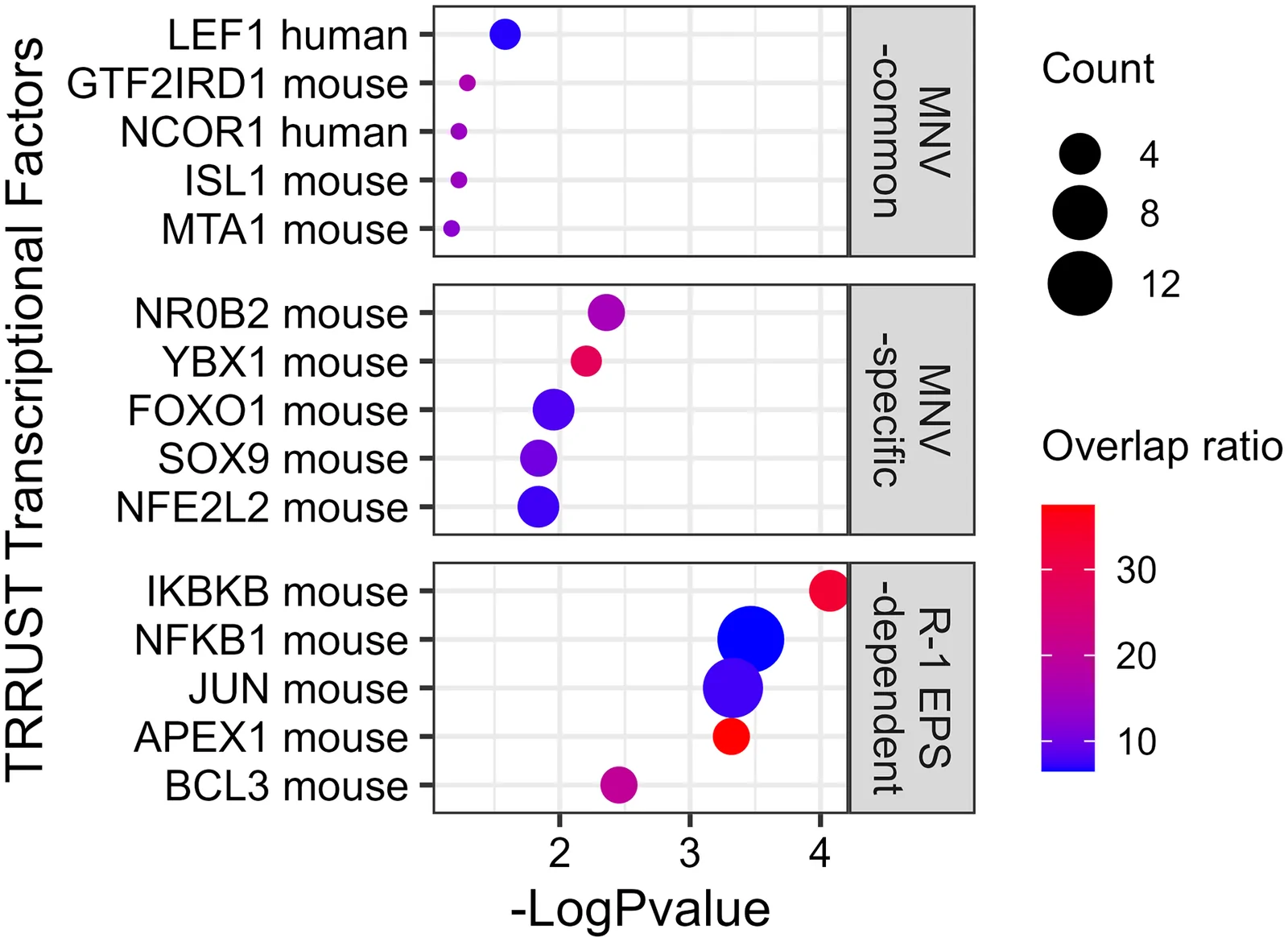
Transcription factor enrichment analysis of overlapping and group-specific differentially expressed genes (DEGs) in MNV-infected cells. Upstream transcription factor enrichment analysis was conducted for three DEG sets: MNV-specific DEGs, defined as DEGs upregulated exclusively in the MNV group; MNV-common DEGs, defined as DEGs upregulated in both the MNV and MNV + R-1 EPS group; and R-1 EPS-dependent DEGs, defined as genes upregulated uniquely in the MNV + R-1 EPS group. Results are shown as a bubble plot, where the *x*-axis represents the -log_10_ *P*-value for each transcription factor. The top five transcription factors ranked by *P*-value for each DEG set are shown. The color of each bubble indicates the overlap ratio (the proportion of genes annotated to each transcription factor within the DEG set), and the bubble size corresponds to the number of enriched genes associated with each transcription factor.

Subsequently, functional enrichment analysis was conducted on the three distinct DEG sets. First, an upstream transcription factor analysis for each DEG set was conducted to identify the potential regulators driving these transcriptional programs (Fig 3). The R-1 EPS-dependent DEGs was enriched for transcription factors involved in inflammatory and antiviral immune responses, including IKBKB, NFKB1, JUN, and APEX1. This enrichment suggests that R-1 EPS-dependent transcriptional changes were associated with the antiviral and inflammatory response pathways during MNV infection.

The MNV-common DEGs exhibited enrichment of transcriptional regulators, such as LEF1 and GTF2IRD1, which are associated with transcriptional regulation and chromatin-related processes [51–53], and in the MNV-specific DEGs, transcriptional regulators related to cellular metabolism and stress responses, such as NR0B2, YBX1, and FOXO1 [54–56], were identified as potential upstream regulators. However, the statistical significance of their enrichment was modest relative to that observed in the MNV + R-1 EPS DEGs, suggesting a less specific and more generalized association with virus-induced stress-adaptive transcriptional programs. Moreover, these transcription factors have been reported to play a role in suppressing inflammatory and antiviral responses [57,58] as well as promoting viral replication [59]. Therefore, MNV may modulate host immune responses in macrophages to facilitate its own replication.

**Fig 4:**
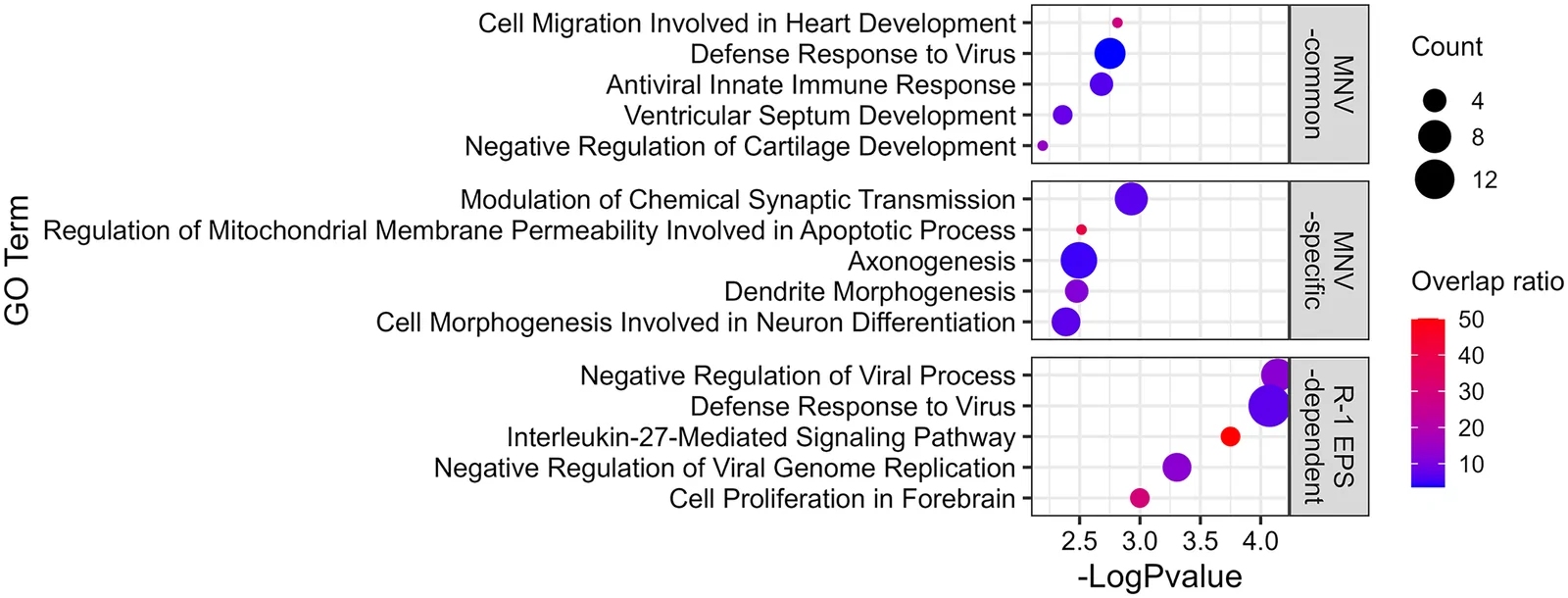
Enriched Gene Ontology (GO) terms of overlapping and group-specific DEGs in MNV-infected cells. Gene ontology analysis was conducted for three DEG sets: MNV-common, MNV-specific, and R-1 EPS-dependent DEGs. Results are shown as a bubble plot, where the *x*-axis represents -log_10_ *P*-value for each GO term. The top five GO terms ranked by *P*-value for each DEG set are shown. The color of each bubble indicates the overlap ratio (the proportion of genes annotated to each GO term within the DEG set), and the bubble size corresponds to the number of enriched genes associated with each GO term.

To characterize the biological processes associated with each DEG set, Gene Ontology (GO) enrichment analysis was conducted (Fig 4). The R-1 EPS-dependent DEGs exhibited enrichment of GO terms directly related to antiviral activity, including “Negative regulation of viral process” and “Defense response to virus,” suggesting a tendency toward selective activation of antiviral biological processes upon R-1 EPS treatment during MNV infection. GO terms associated with antiviral responses, such as “defense response to virus” and “antiviral innate immune response,” were also detected in the MNV‑common DEGs; however, the *P*‑values were higher and the number of genes annotated to these GO terms was smaller than those in the R-1 EPS‑dependent DEGs, indicating weaker enrichment in the MNV‑common DEGs. In the MNV-common DEGs, GO terms related to antiviral responses as well as developmental and structural processes were identified, suggesting broadly-distributed and non-specific transcriptional changes induced by MNV infection, irrespective of R-1 EPS treatment. GO terms associated with neuronal morphogenesis, synaptic modulation, and mitochondrial regulation were detected in the MNV-specific DEGs, suggesting diverse transcriptional changes induced by MNV infection that lacked a unified functional theme.

**Fig 5:**
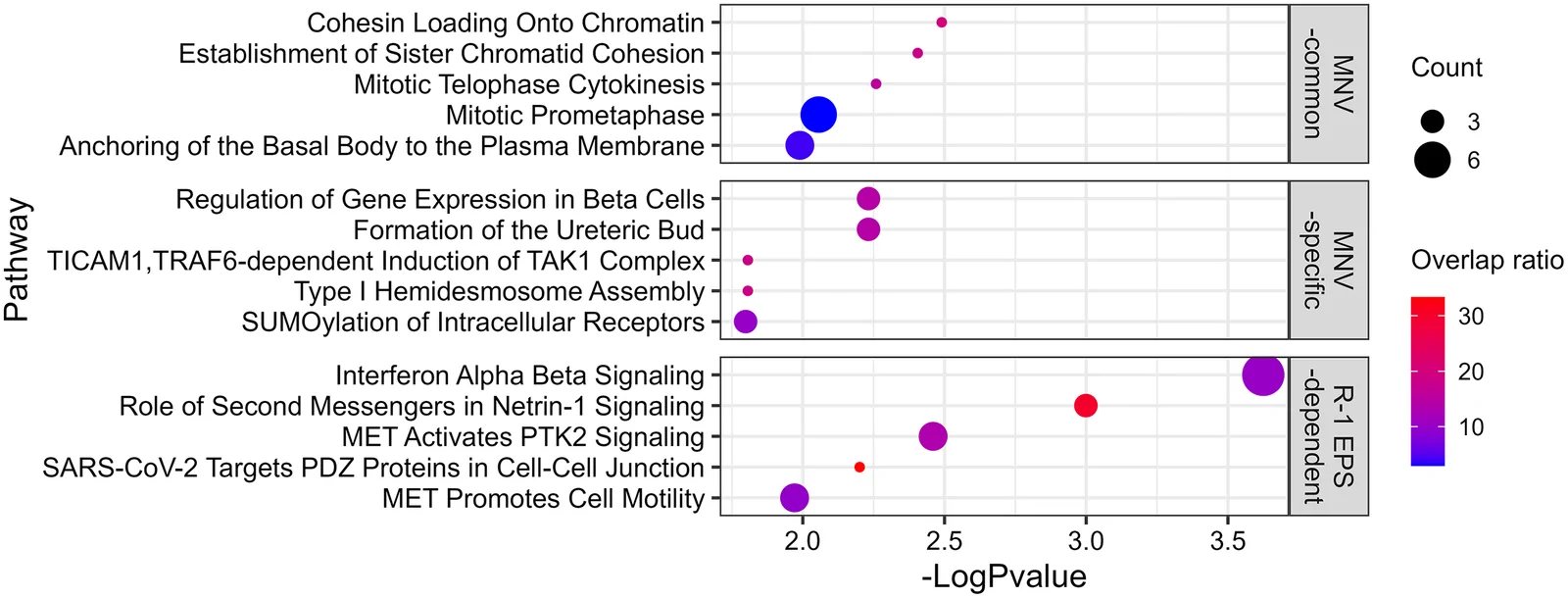
Enriched pathways of overlapping and group-specific DEGs in MNV-infected cells. Pathway enrichment analysis was conducted for three DEG sets: MNV-common, MNV-specific, and R-1 EPS-dependent DEGs. Results are shown as a bubble plot, where the *x*-axis represents -log_10_ *P*-value for each pathway. The top five pathways ranked by *P*-value for each DEG set are shown. The color of each bubble indicates the overlap ratio (the proportion of genes annotated to each pathway within the DEG set), and the bubble size corresponds to the number of enriched genes associated with each pathway.

To investigate the signaling pathways associated with each DEG set, a pathway enrichment analysis was conducted (Fig 5). In the R-1 EPS-dependent DEGs, interferon alpha/beta signaling was highlighted at the pathway level, consistent with the GO enrichment analysis, indicating that R-1 EPS treatment preferentially engaged interferon-mediated antiviral responses during MNV infection. In the MNV-common DEGs, pathway analysis highlighted processes related to cell cycle progression and chromatin dynamics, including sister chromatid cohesion and mitotic events, suggesting broad alterations in fundamental cellular processes induced by MNV infection, irrespective of R-1 EPS treatment. In the MNV-specific DEGs, pathway analysis identified various pathways related to development, signaling, and cellular structural regulation, suggesting heterogeneous transcriptional changes that did not converge on a specific signaling pathway uniquely induced by MNV infection.

**Fig 6:**
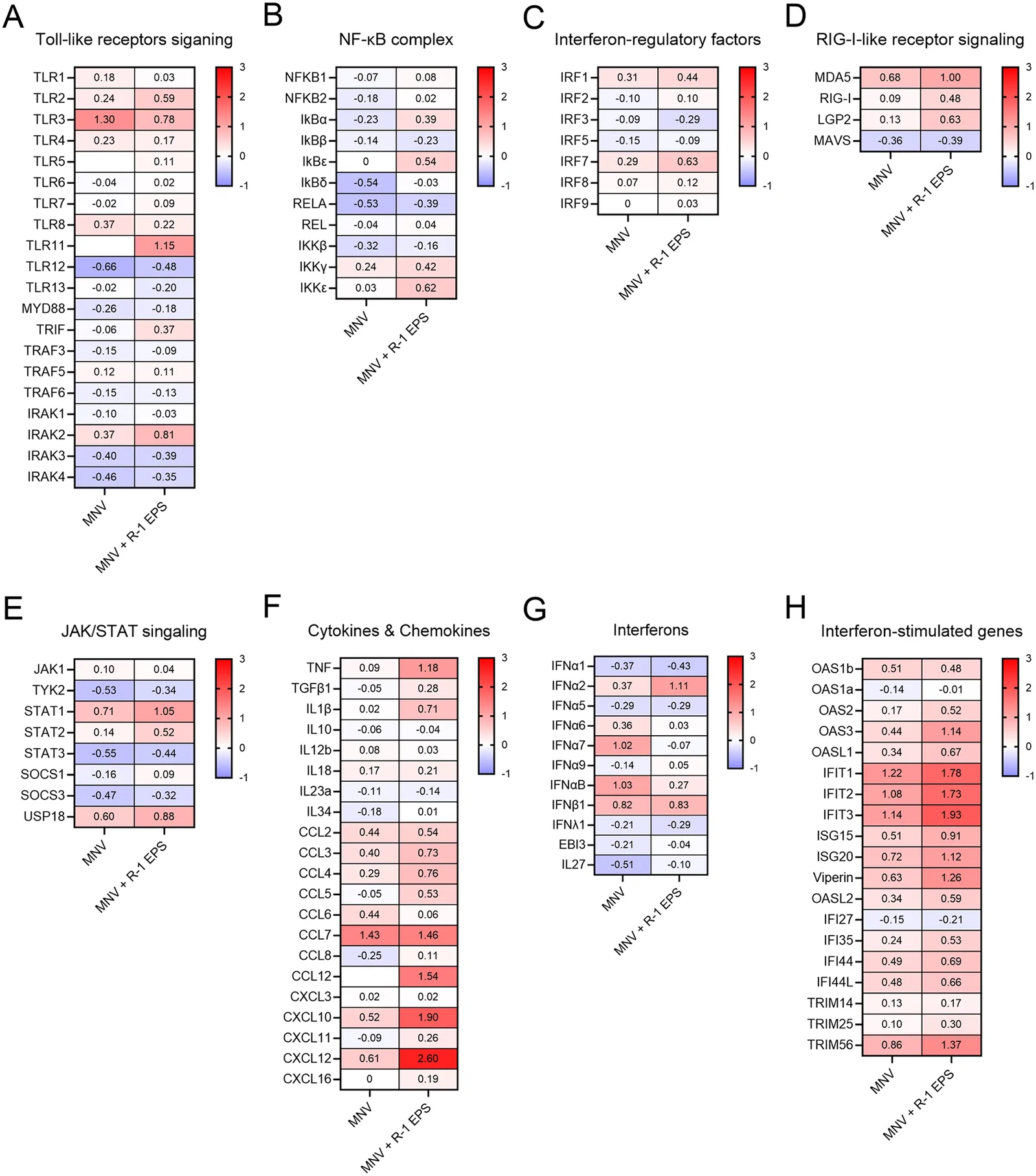
Gene expression of signaling pathways involved in inflammatory and antiviral responses. Gene expression is shown as log_2_ fold‑change (log_2_FC) heatmaps calculated for the MNV and MNV + R-1 EPS groups, each normalized to non‑infected cells. Shown are the gene expression profiles of: (A) Toll-like receptor signaling components, (B) NF-κB complex, (C) IFN-regulatory factors, (D) RIG-I-like receptor signaling, (E) JAK/STAT signaling components, (F) Cytokines and chemokines, (G) Interferons, and (H) IFN-stimulated genes.

Based on the preceding analyses, including upstream transcription factor, GO, and pathway enrichment analyses, R-1 EPS treatment was suggested to enhance the antiviral and inflammatory responses upon MNV infection. To investigate this possibility, the activation status of major signaling pathways involved in antiviral and inflammatory responses were evaluated by comparing gene expression levels between the MNV and MNV + R-1 EPS groups. Genes associated with key innate immune and antiviral pathways, including TLR, IRF, RLR signaling; NF‑κB complex; and the JAK/STAT pathway, were generally expressed at higher levels in the MNV + R-1 EPS compared to the MNV group (Fig 6A–6E). Increased expression of cytokines and chemokines was observed in response to the activation of these upstream pathways (Fig 6F). Although the expression of some type I IFN genes was reduced in MNV + R-1 EPS group (Fig 6G), the expression levels of multiple ISGs were elevated in these groups (Fig 6H). Because the induction of IFN genes can be transient, changes in IFN expression may not necessarily be detected at the individual gene level. However, the enhanced expression of several downstream ISGs suggested that IFN responses were induced in the MNV + R-1 EPS group. Taken together, these results indicate that antiviral and inflammatory responses were concomitantly enhanced in the MNV + R-1 EPS group.

**Fig 7:**
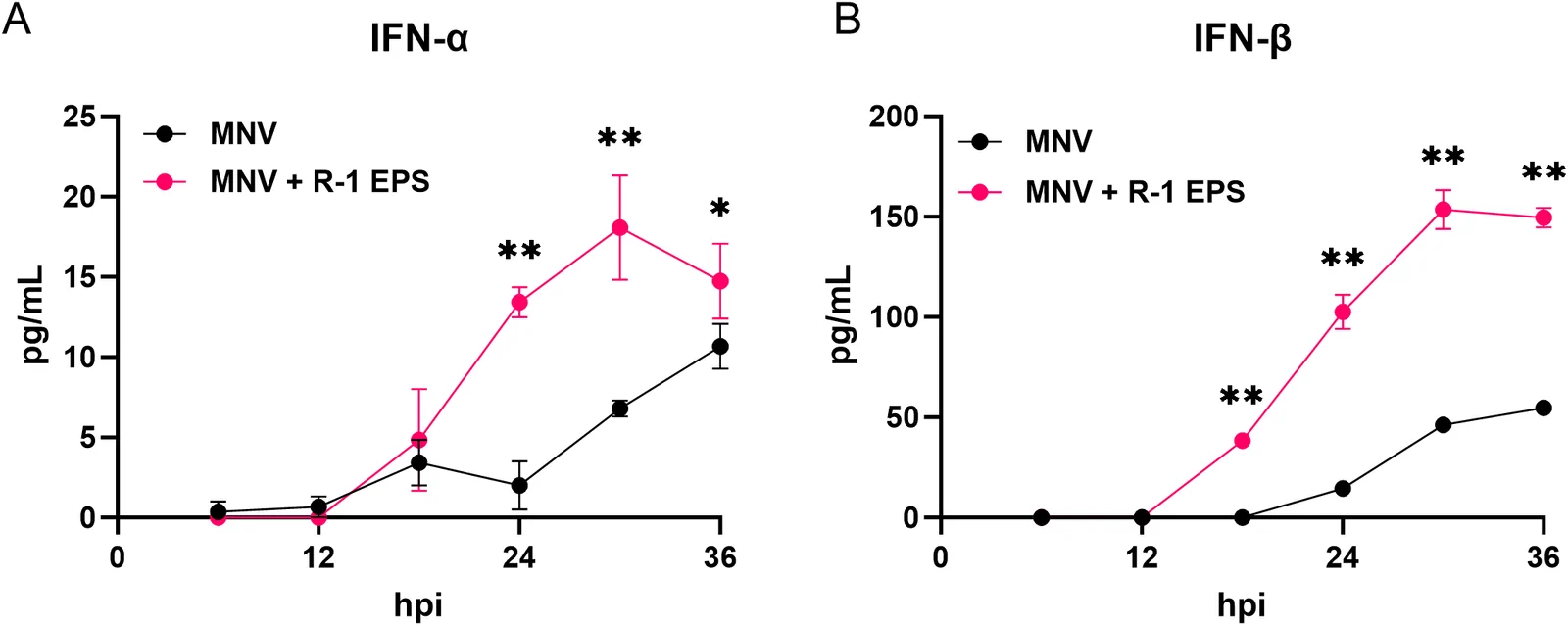
R-1 EPS upregulated type I IFN expression in MNV-infected cells. RAW264.7 cells were infected with MNV-S7 and subsequently treated with 200 µg/mL R-1 EPS (MNV + R-1 EPS). Cells infected and cultured without R-1 EPS were used as untreated control (MNV). The cell culture supernatant was collected every 6 h. (A) IFN-α and (B) IFN-β levels were quantified using enzyme-linked immunosorbent assay (ELISA). Black circles represent untreated samples and magenta circles represent R-1 EPS-treated samples. Data represent mean ± standard deviation (SD) of triplicate wells per group. Two independent biological replicates were conducted and representative results are shown. *: *P* < 0.05, **: *P* < 0.01 (two-way ANOVA followed by Sidak’s post-hoc test vs. the corresponding control).

Microarray-based transcriptomic analysis suggested that antiviral and inflammatory responses were enhanced in the MNV + R-1 EPS group. To validate these findings, we investigated whether type I IFN production, ISG induction, and inflammatory responses were induced in MNV + R-1 EPS-treated cells. Culture supernatants were collected every 6 h from infected RAW264.7 cells, and the IFN-α and β levels were quantified. IFN-α secretion was significantly higher in the MNV + R-1 EPS group at 24, 30, and 36 hpi compared to the MNV group (Fig 7A). IFN-β levels were also significantly elevated in the MNV + R-1 EPS group starting at 18 hpi (Fig 7B). When uninfected cells were stimulated with R-1 EPS alone, both IFN-α and IFN-β were below the detection limit of the assay (S3 Fig). These results suggest that R-1 EPS enhanced type I IFN production during MNV infection.

**Fig 8:**
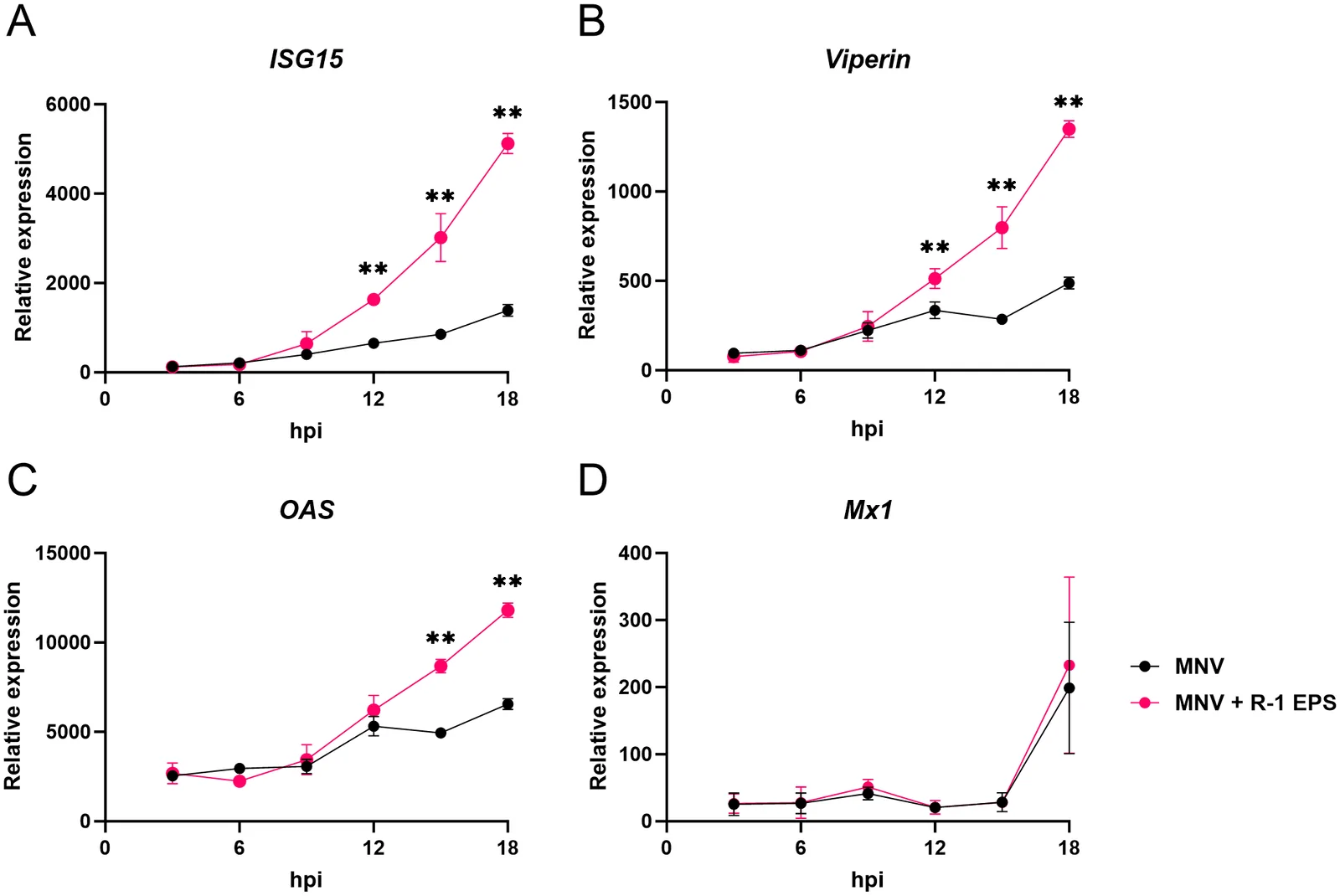
R-1 EPS upregulated the expression of interferon-stimulated genes (ISGs) in MNV-infected cells. RAW264.7 cells were infected with MNV-S7 and subsequently treated with 200 µg/mL R-1 EPS (MNV + R-1 EPS). Cells infected and cultured without R-1 EPS were used as untreated control (MNV). RNA was isolated from the cells collected every 3 h for quantifying the expression of (A) *ISG15*, (B) *Viperin*, (C) *OAS*, and (D) *Mx1* using RT-qPCR. Gene expression levels are presented as relative expression levels, normalized to hypoxanthine phosphoribosyltransferase (*HPRT*) expression with an expression level set to 10,000. Black circles represent the MNV group and magenta circles represent the MNV + R-1 EPS group. Data represent mean ± standard deviation (SD) of triplicate wells per group. The experiment was conducted as a single biological replicate. **: *P* < 0.01 (two-way ANOVA followed by Sidak’s post-hoc test vs. each control).

Because type I IFNs exert their antiviral effects through the induction of ISGs, the expression of representative ISGs were determined using RT-qPCR. The expression of *ISG15* and *Viperin* was significantly higher in the MNV + R-1 EPS group at 12, 15, and 18 hpi than in the MNV group (Fig 8A, 8B). The expression of 2′-5′-oligoadenylate synthetases (*OAS*) was significantly higher in the MNV + R-1 EPS group at 15 and 18 hpi compared to the MNV group (Fig 8C). In contrast, the expression of MX dynamin-like GTPase 1 (*Mx1*) was not significantly different between the MNV and MNV + R-1 EPS groups (Fig 8D). These findings indicate that the inhibitory effect of R-1 EPS is associated with the enhancement of IFN signaling and the subsequent induction of ISGs following MNV infection.

**Fig 9:**
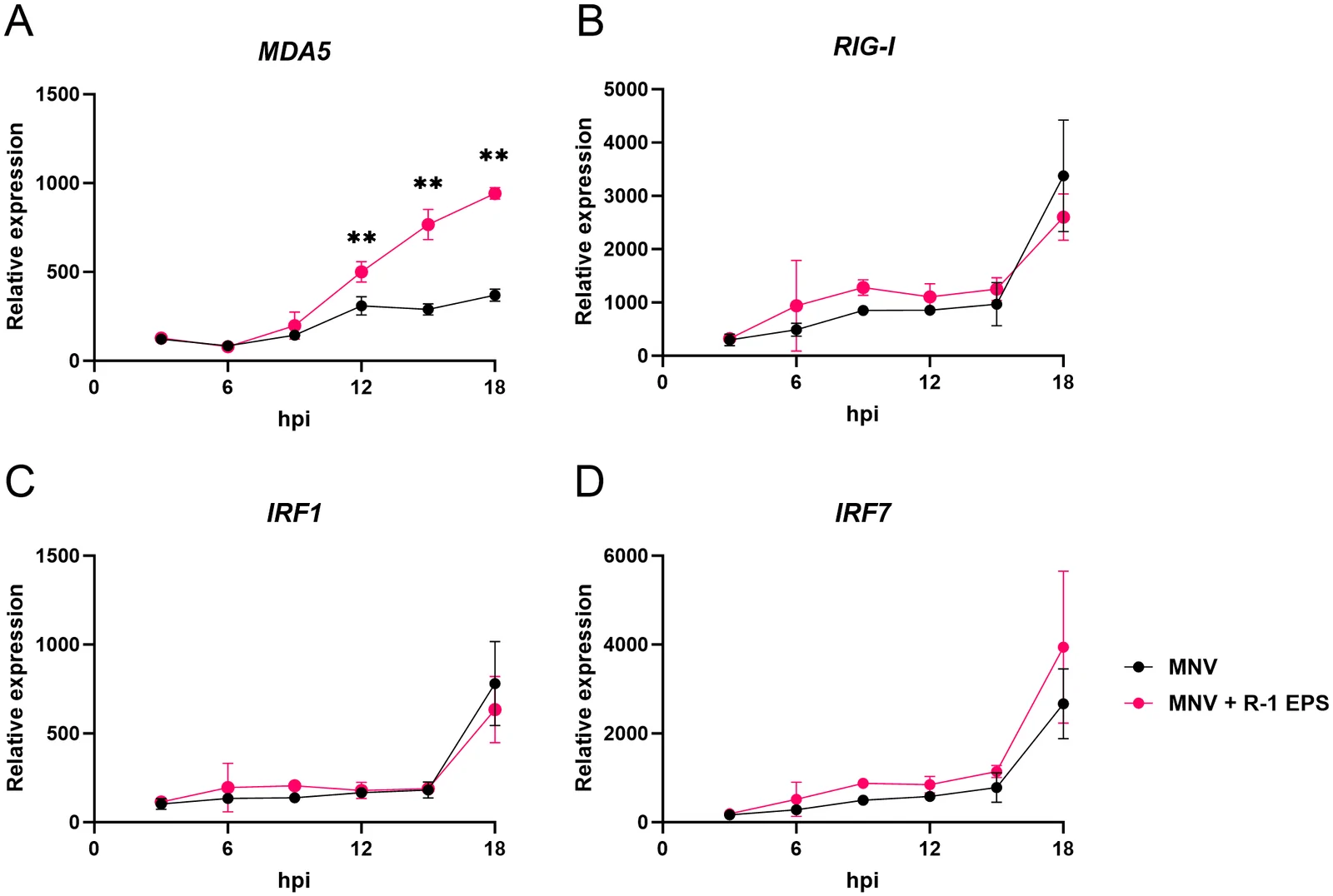
R-1 EPS upregulated gene expression of signal transduction factors inducing IFN expression in MNV-infected cells. RAW264.7 cells were infected with MNV-S7 and subsequently treated with 200 µg/mL R-1 EPS (MNV + R-1 EPS). Cells infected and cultured without R-1 EPS were used as untreated control (MNV). RNA was isolated from the cells collected every 3 h for quantification of (A) *MDA5*, (B) *RIG-I*, (C) *IRF1*, and (D) *IRF7* expression using RT-qPCR. Gene expression levels are presented as relative expression levels, normalized to *HPRT* expression with an expression level set to 10,000. Black circles represent the MNV and magenta circles represent the MNV + R-1 EPS group. Data represent mean ± SD of results from two or three replicate wells per group. The experiment was conducted as a single biological replicate. **: *P* < 0.01 (two-way ANOVA followed by Sidak’s post-hoc test vs. each control).

To further elucidate the mechanism underlying the induction of type I IFNs by R-1 EPS, we next examined upstream signaling molecules involved in IFN production. Melanoma differentiation-associated protein 5 (*MDA5*) and *RIG-I* are cytoplasmic sensor proteins involved in the detection of viral RNA and in triggering type I IFN signaling. However, in our study, only *MDA5* expression was significantly upregulated in the MNV + R-1 EPS group at 12, 15, and 18 hpi compared to the MNV group (Fig 9A, 9B). This increase in MDA5 expression may contribute to the increase in IFN-α and -β production in the late stages of infection, from 18 hpi onward. In contrast, the expression of *IRFs*, which are downstream molecules of pattern recognition receptors and function as transcription factors that activate IFN gene expression, showed no significant difference in the MNV and the MNV + R-1 EPS groups (Fig 9C, 9D). Although not all genes involved in interferon-related signaling pathways showed significant upregulation at the transcript level using RT‑qPCR, the consistent induction of multiple ISGs, together with the increased production of IFN‑α and IFN‑β at the protein level (Fig 7), suggests functional activation of interferon‑mediated antiviral responses in the MNV + R-1 EPS group.

**Fig 10:**
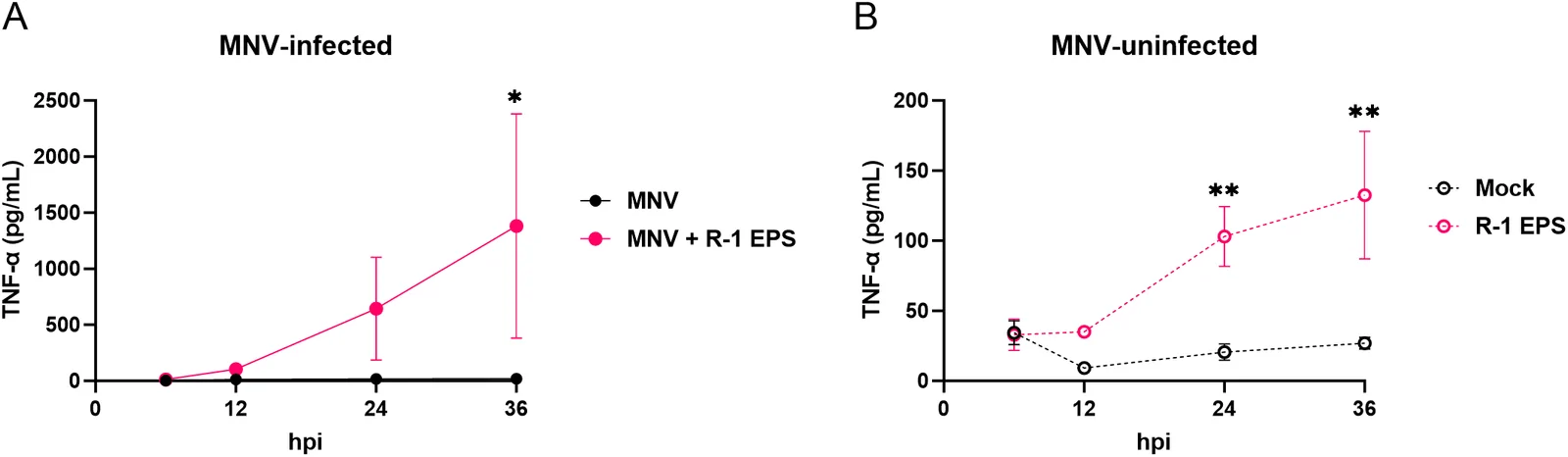
R-1 EPS upregulated TNF-α expression in both MNV-infected and uninfected cells. (A) RAW264.7 cells were infected with MNV-S7 and subsequently treated with 200 µg/mL R-1 EPS (MNV + R-1 EPS). Cells infected and cultured without R-1 EPS were used as untreated control (MNV). (B) Uninfected RAW264.7 cells were cultured without or with 200 µg/mL of R-1 EPS. In both infected and uninfected conditions, the cell culture supernatant was collected at 6, 12, 24, and 36 h. TNF-α levels were quantified using enzyme-linked immunosorbent assay (ELISA). Black circles represent untreated samples and magenta circles represent R-1 EPS-treated samples. Data represent mean ± standard deviation (SD) of triplicate wells per group. Two independent biological replicates were conducted and representative results are shown. *: *P* < 0.05, **: *P* < 0.01 (two-way ANOVA followed by Sidak’s post-hoc test vs. the corresponding control).

Whether R-1 EPS treatment modulated the inflammatory response during MNV infection was investigated. TNF‑α is proinflammatory cytokine that is reported to be induced upon MNV infection [60]. Upstream regulator analysis of the microarray data identified NF‑κB signaling as a prominently-activated pathway in the MNV + R-1 EPS group (Fig 3) and TNF‑α, a canonical downstream target of NF‑κB, was therefore the focus of the investigation. TNF‑α production was significantly increased in the MNV + R-1 EPS compared to the MNV group (Fig 10A). To determine whether R-1 EPS treatment affected TNF‑α production in the absence of viral infection, TNF‑α levels were also measured in uninfected cells. Under non‑infected conditions, R-1 EPS treatment was sufficient to induce TNF‑α production (Fig 10B). However, the absolute level of TNF‑α induced by R-1 EPS alone was approximately tenfold lower than that observed during MNV infection. These results suggest that R-1 EPS treatment enhanced TNF‑α production predominantly in the context of MNV infection.

Other proinflammatory cytokines were examined using multiplex analysis. IL-6, MCP-1, MIP-1α, and MIP-1β levels were significantly upregulated in the MNV + R-1 EPS compared to the MNV group (S4 Fig). IL-1α, IL-1β, IL-12, and KC were also measured but were below the detection limit at all time points in both groups.

**Fig 11:**
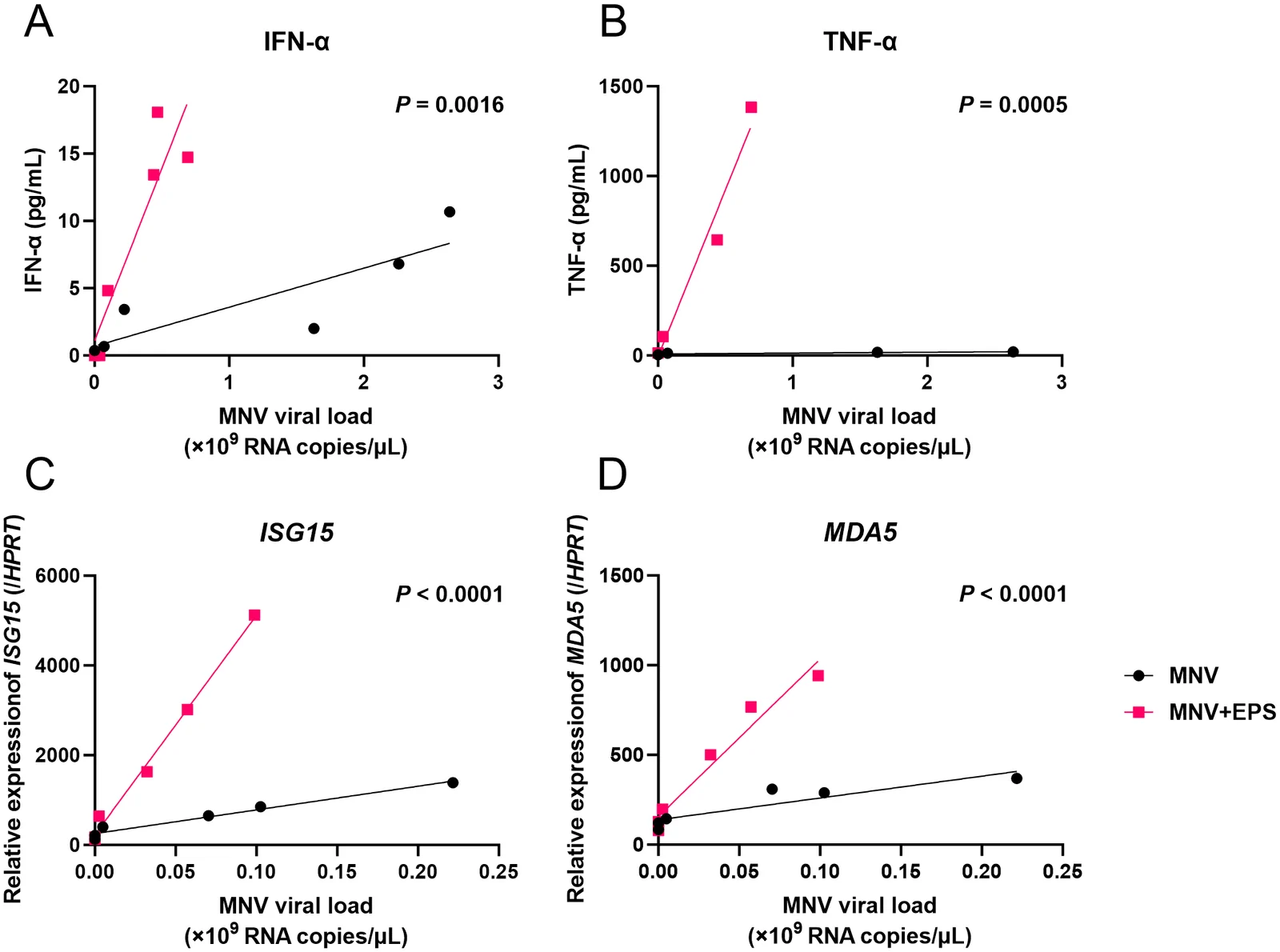
Relationship between viral load and host responses in the presence or absence of R-1 EPS. Scatter plots showing the relationship between MNV viral load and host antiviral responses, including cytokine protein levels and antiviral gene expression, in the presence or absence of R-1 EPS. Each point represents an individual time point. Solid lines indicate regression fits for each group. The *P*-value indicates the statistical significance of the difference in the relationship between viral load and host antiviral responses in the presence vs. the absence of R-1 EPS. The presence of R-1 EPS is associated with an altered relationship between viral replication and host antiviral responses, suggesting that R-1 EPS modifies host antiviral signaling relative to viral burden.

Both the viral load and host cytokine and ISG responses changed dynamically over the course of infection. To understand how these responses were related to viral replication, the relationship between viral load and host antiviral responses was investigated. Although increases in cytokine and ISG expression were observed in parallel with viral replication in both groups, R-1 EPS treatment appeared to modulate the magnitude of host antiviral responses relative to the viral burden (Fig 11, S5 Fig). These results suggest that R-1 EPS affected the interaction between viral replication and host antiviral signaling, potentially contributing to the observed suppression of MNV infection.

**Fig 12:**
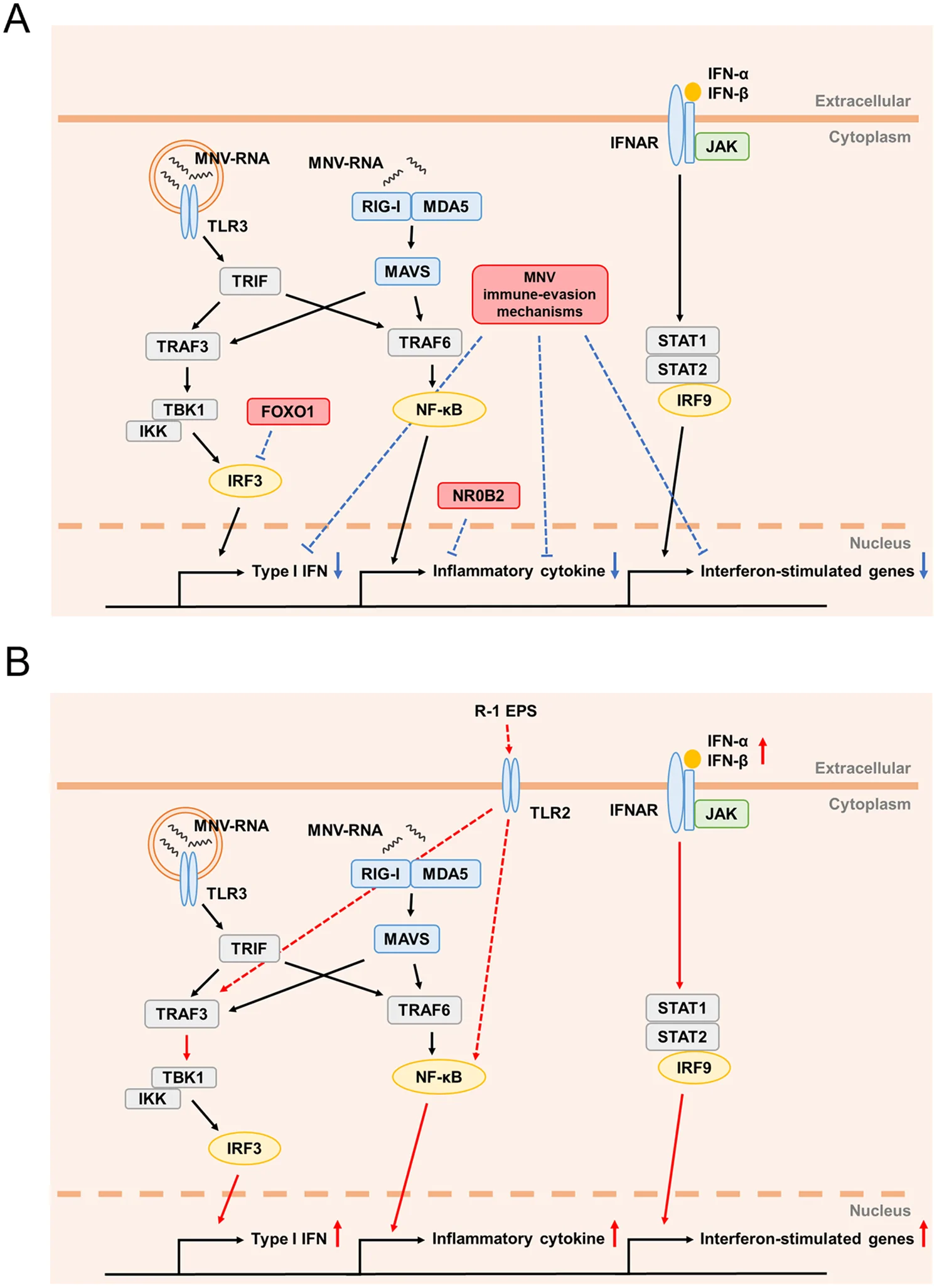
Proposed schematic model of R-1 EPS effects on host innate immune responses during MNV infection. The model integrates the experimental findings of this study, including transcriptional activation, and cytokine production, together with previously-reported signaling pathways. Red arrows indicate signaling pathways for which R-1 EPS was suggested to enhance host responses. Dashed lines represent mechanisms inferred from prior literature.

Overall, our results suggest that treatment of R-1 EPS after MNV infection activates the innate immune response, especially type I IFN signaling, which is associated with reduced MNV proliferation. The proposed mechanisms of R-1 EPS in the host innate immune response during MNV infection are summarized in Fig 12. As shown in the transcriptome analysis, enrichment of transcription factors, such as NR0B2 and FOXO1, which are associated with the suppression of inflammatory and antiviral responses, was observed in the MNV-specific group (Fig 3). NR0B2 has been reported to act as a negative regulator of inflammatory responses in macrophages [58], whereas FOXO1 suppresses type I interferon production by suppressing the activity of IRF3 [57]. MNV suppresses host IFN production and inflammatory responses [27,61]. The current results suggest that R-1 EPS treatment enhances host immune responses and modulates the suppressed antiviral pathways, thereby contributing to the inhibition of MNV replication.

## Discussion

Norovirus has different genotypes and evolves quickly, making it difficult to design a vaccine that provides broad-spectrum and lasting protection because new variants can quickly render existing vaccines less effective [62]. Therefore, developing defense strategies that target the host innate immunity may be effective, as they have the potential to provide broad-spectrum protection against norovirus. In this study, we demonstrated the efficacy of R-1 EPS in suppressing MNV infection by enhancing the type I IFN response and the potential of R-1 EPS as a functional food ingredient effective in enhancing innate immunity against infection.

Recent studies have proposed *in vitro* culture models using human enteroids for HuNoV; however, there is a lack of efficient *in vitro* cell culture methods and easily usable animal models for HuNoV cultivation [9]. Therefore, numerous studies have used MNV as a surrogate for HuNoV [10]. MNV has a tropism for macrophages and dendritic cells and replicates robustly in RAW264.7 cells [8]; therefore, we employed this culture system in this study.

In this study, R-1 EPS demonstrated a maximum inhibition rate of 79.3% against MNV (S1 Table). Previous reports have shown that other natural compounds exhibiting comparable levels of *in vitro* inhibition (approximately 80–90%) were later confirmed to reduce viral load or disease severity *in vivo*. A glycolipid isolated from the microalga *Coccomyxa* sp. KJ exhibited approximately 90% inhibition of MNV *in vitro* and subsequently suppressed viral shedding in feces following oral administration in mice [63]. Similarly, fucoidan derived from brown algae, *Laminaria japonica*, demonstrated approximately 85–95% inhibition of MNV *in vitro* and significantly improved survival and reduced fecal viral titers in MNV-infected mice [64]. Although the magnitude of viral suppression observed *in vitro* cannot be directly extrapolated to *in vivo* efficacy, the *in vitro* findings of our study showed a consistent induction of antiviral transcriptional responses within the experimental system. Therefore, further studies are required to determine whether R-1 EPS exerts inhibitory effects against MNV *in vivo*.

Gene expression analysis using microarray and other quantitative analyses revealed enhanced type I IFN signaling in MNV-infected cells treated with R-1 EPS. These findings suggest that the enhancement of type I IFN signaling post-R-1 EPS treatment associated with reduced MNV replication. The importance of type I IFN signaling in the antiviral response is well known and it is also reported to be effective in MNV and HuNoV replication. In mice with a myeloid cell-specific knockout of IFN-α/β receptor, MNV load was increased in tissues including the intestine [22]. IFN-β is reported to suppress RNA replication and protein expression of HuNoV replicon in human cell lines [65]. The antiviral effectors that directly suppress MNV and HuNoV replication are not fully understood, although some ISGs have been reported to inhibit MNV and HuNoV replication *in vivo* and *in vitro*. ISG15 has been reported to suppress MNV replication both *in vivo* and *in vitro* [66] and Viperin is suggested to be an effective factor in the suppression of HuNoV replication, as its expression is upregulated following infection [67]. MDA5, whose expression was upregulated in R-1 EPS-treated cells following infection in this study (Fig 4), has been reported to inhibit the replication of HuNoV replicons [68], thereby serving a potent suppressive factor against HuNoV. These results suggest that the enhancement of type I IFN signaling by R-1 EPS contributes to the suppression of MNV replication. While type I IFN responses play a pivotal role in restricting MNV and HuNoV replication, these viruses have evolved strategies to counteract this antiviral pathway. It has been reported that MNV proteins have the ability to suppress type I IFN production in host cells, thereby establishing efficient infection [61,69]. Nonstructural proteins of HuNoV have also been suggested to suppress type I IFN production [70–74]. Therefore, the ability of R-1 EPS to enhance the type I IFN signaling ever after infection may help overcome these viral immune evasion mechanisms, representing a promising strategy for preventing MNV and HuNoV infection. Our microarray analysis of several IFN subtype genes showed that R-1 EPS treatment increased the expression of some IFN-α subtypes but decreased that of others compared with MNV infection alone. In particular, IFN-α2 expression increased, whereas IFN-αB and IFN-α7 expression decreased relative to MNV infection alone (Fig 6G). Although these findings raise the possibility that R-1 EPS may differentially affect the expression of individual IFN-α subtypes, the present microarray analysis was limited to a single time point and included only a subset of IFN-α subtype genes. Therefore, it remains unclear whether the observed differences reflect subtype-specific regulation by R-1 EPS or differences in the induction kinetics of individual IFN-α subtypes. Importantly, our ELISA quantified total IFN-α protein across all subtypes (Fig 7A), and several ISGs induced downstream of type I IFN signaling were also upregulated in the MNV + R-1 EPS group (Fig 6H, Fig 8). Therefore, we consider the overall enhancement of IFN production to be most relevant to the antiviral response observed in this study.

Upstream transcription factor analysis based on the microarray data identified NR0B2, YBX1, and FOXO1 as putative regulators only in MNV-infected cells and not in R-1 EPS-treated, MNV-infected cells. These observations suggest that MNV infection may exploit these regulatory pathways to attenuate host inflammatory and antiviral responses, thereby creating a cellular environment that favors viral replication. In contrast, treatment with R-1 EPS was associated with the absence of these immunosuppressive transcriptional signatures, suggesting that R-1 EPS may counteract the virus-induced suppression of host responses. By counteracting the virus-associated suppression of inflammatory and antiviral signaling pathways, R-1 EPS may enhance host-mediated control of MNV replication, contributing to the observed reduction in viral load. On the other hand, the ability of R-1 EPS to promote type I IFN production may be influenced by cell type and the intestinal environment. Therefore, further studies using enteroids for HuNoV and *in vivo* mouse models are necessary.

Although the precise mechanisms by which R-1 EPS enhances type I IFN responses remain unclear, Kanmani *et al*. reported that co-stimulation with R-1 EPS and poly(I:C) upregulates the expression of type I IFN genes in porcine epithelial cells [41]. During MNV replication, double-stranded RNA (dsRNA) can be formed as an intermediate in infected cells and be recognized by innate sensors such as TLR3, leading to type I IFN production. Type I IFN was not induced when uninfected cells were treated with R-1 EPS. Instead, our findings revealed that R-1 EPS enhances the type I IFN signaling induced by MNV infection. Notably, Kanmani *et al*. also reported that inhibition of TLR2 suppresses the enhancement of type I IFN expression induced by co-stimulation with R-1 EPS and Poly(I:C) [41]. The specific receptors through which R-1 EPS exerts its immunostimulatory effects remain unclear. However, it has been reported that the immunostimulatory effects of R-1 EPS were absent in myeloid differentiation factor 88 (MyD88) knockout mice, indicating that R-1 EPS may induce signaling via TLR2 or other TLRs [38]. TLR2 signaling predominantly activates NF‑κB–dependent inflammatory pathways and induces cytokines such as TNF‑α, whereas it elicits only limited type I IFN responses when stimulated alone [75]. However, during viral infection, TLR2 signaling cooperates with nucleic acid-sensing PRRs to enhance IRF3‑dependent interferon production [76]. Consistent with this model, the current data demonstrate that R-1 EPS induced TNF‑α (Fig 10) but failed to trigger type I IFN (S3 Fig) production in non‑infected cells, supporting the notion that TLR2 signaling alone was insufficient to drive robust IFN responses. Taken together, R-1 EPS may act as a TLR2 agonist and modulate TLR2- and MyD88-mediated signaling, resulting in the enhancement of type I IFN production triggered by the sensing of dsRNA by TLR3 during viral infection.

Treatment with R-1 EPS promoted the production of proinflammatory cytokines (Fig 10, S4 Fig). TNF-α, whose expression was significantly upregulated in R-1 EPS-treated cells (Fig 10A), has been reported to induce the production of ISGs via TNF receptor independent of type I IFN and the JAK/STAT pathway, and to suppress hepatitis virus replication [77]. TNF-α upregulation by R-1 EPS might contribute to MNV growth inhibition beyond type I IFN signaling.

The inhibitory effect of R-1 EPS on HuNoV replication in intestinal epithelial cells, which are considered the host cells of HuNoV *in vivo*, has not been evaluated. In contrast, Kawanabe *et al.* reported that oral administration of R-1 EPS to mice resulted in the activation of T cells in Peyer’s patch, suggesting that R-1 EPS has the ability to activate intestinal immunity [78]. Moreover, the dietary intake of yogurt fermented with *L. delbrueckii* ssp. *bulgaricus* OLL1073R-1, which contains R-1 EPS, reportedly activated natural killer cells [79], conventional dendritic cells, and plasmacytoid dendritic cells [80] in peripheral blood in clinical studies. These findings suggest that the interaction between R-1 EPS and immune cells within the intestinal mucosa may modulate systemic immune responses.

## Limitation

This study has several limitations. First, the concentration of R-1 EPS used may exceed levels that can be achieved by oral intake *in vivo*, making it unclear whether similar effects would be observed in physiological conditions. Second, because our data were obtained from *in vitro* experiments using RAW264.7 cells infected with MNV, it does not fully represent the gastrointestinal environment where human norovirus infects. Differences in cell tropism, receptor usage, and innate immune responses between this murine macrophage system and the human intestinal epithelium limit direct extrapolation of our findings to clinical settings. Future studies using appropriate animal models, HuNoV infection systems, and clinical trials will be necessary to verify the physiological relevance and antiviral potential of R-1 EPS.

## Conclusion

In conclusion, we demonstrated that exopolysaccharide derived from *L. delbrueckii* ssp. *bulgaricus* OLL1073R-1 (R-1 EPS) promotes type I IFN signaling and proinflammatory cytokine production following MNV infection *in vitro* model and suppresses MNV replication. These findings provide mechanistic insight into host-directed antiviral effect of microbial exopolysaccharides. Further *in vivo* and clinical studies are required to validate its physiological relevance.

## Supporting information

S1 Fig WST-1 cell proliferation assay of R-1 EPS-treated cells. Viability of RAW264.7 cells cultured without or with 200 µg/mL of R-1 EPS for 12 h or 36 h was measured using the WST-1 assay. Absorbance at 450 nm and 690 nm was measured at 4 h after the addition of WST-1 reagent to RAW264.7 cells. Data are shown as mean ± SD (n = 3). nd: no significant difference was detected between each group by Student’s t-test.
(TIF)

S2 Fig Assessment of direct virucidal effects of R-1 EPS on MNV. MNV was pre-incubated without (untreated MNV) or with 200 µg/mL of R-1 EPS (R-1 EPS pre-treated MNV). After incubation, MNV was ultracentrifuged to separate the viral particles from unbound R-1 EPS. The pre-incubated MNV was then used to infect RAW264.7 cells. The culture supernatant was collected every 24 h and viral RNA copy numbers were quantified using RT-qPCR. Black bars represent the untreated MNV group and magenta bars represent the R-1 EPS-pretreated MNV group. Data are presented as the geometric mean ± standard deviation (SD) of three wells per group. nd: no significant difference by Mann–Whitney U test.
(TIF)

S3 Fig Type I IFN expression in uninfected cells stimulated with R-1 EPS. RAW264.7 cells were cultured without or with 200 µg/mL of R-1 EPS for 0, 12, 24, and 36 h. (A) IFN-α and (B) IFN-β levels were quantified using ELISA. The methods and the specific ELISA kits used for quantifying each cytokine are described in the S1 Protocol. According to each protocol, absorbance at 450 nm was measured for the quantification of IFN-α, while absorbance at both 450 nm and 570 nm was measured for the quantification of IFN-β. Left panels show the blank corrected OD value and concentration of the standard of ELISA. Right panels show the blank corrected OD value of each sample. Black circles represent untreated samples and open circles represent R-1 EPS-treated samples. The experiment was performed in triplicate wells for each condition. The OD values of all samples were below the lower limit of quantification of the assay, indicating undetectable levels of both cytokines in the tested samples.
(TIF)

S4 Fig R-1 EPS enhanced inflammatory cytokine production in MNV-infected cells. RAW264.7 cells were infected with MNV-S7 and subsequently treated with 200 µg/mL R-1 EPS (MNV + R-1 EPS). Cells infected and cultured without R-1 EPS served as untreated controls (MNV). The cell culture supernatants were collected every 3 h. The (A) IL-6, (B) TNF-α, (C) MCP-1, (D) MIP-1α, and (E) MIP-1β levels were quantified using a multiplex assay system (Bio-Plex). The MIP-1α level after 18 hpi is not shown because it exceeded the upper limit of quantification. Black and magenta circles represent the MNV and MNV + R-1 EPS groups, respectively. IL-1α, IL-1β, IL-12, and KC were also measured but were below the detection limit at all time points in both groups. Data represent mean ± standard deviation (SD) of results from triplicate wells per group. Each experiment was conducted as a single biological replicate. *: *P* < 0.05, **: *P* < 0.01 (two-way ANOVA followed by Sidak’s post-hoc test vs. each control).
(TIF)

S5 Fig Relationship between viral load and host antiviral responses in the presence or absence of R-1 EPS. Scatter plots showing the relationship between MNV viral load and host antiviral responses, including cytokine protein levels and antiviral gene expression, in the presence and absence of R-1 EPS. Data not shown in Fig 11 are presented here. Each point represents an individual time point. The solid lines indicate the regression fit for each group. The *P*-value indicates the statistical significance of the difference in the relationship between viral load and host antiviral responses in the presence and absence of R-1 EPS.
(TIF)

S1 Table Inhibition rate of R-1 EPS treatment in MNV-infected RAW264.7. (DOCX)

S1 Data Raw data of the microarray analysis. (XLSX)

S1 Protocol Supplementary methods. (DOCX)
