# Supplementary material for: Lactobacillus delbrueckii ssp. bulgaricus OLL1073R-1-derived exopolysaccharide suppresses murine norovirus replication by modulating innate immune response
Source: PLoS One. 2026 Sep 2;21(9):e0356886. doi: 10.1371/journal.pone.0356886 (PMC13537558; doi:10.1371/journal.pone.0356886)
Supplement: Supplementary file 1 [file pone.0356886.s006.docx]

**S1 Table. Inhibition rate of R-1 EPS treatment in MNV-infected RAW264.7.**

| hpi | Viral load (×10^9^ RNA copies/µL) | | | | | | |  | Inhibition rate (%) |
| --- | --- | --- | --- | --- | --- | --- | --- | --- | --- |
|  | Control | | |  | R-1 EPS | | |  |  |
| 0 | 0.02 | ± | 0.00 |  | 0.02 | ± | 0.00 |  | -4.3 |
| 3 | 0.00 | ± | 0.00 |  | 0.00 | ± | 0.00 |  | 44.4 |
| 6 | 0.00 | ± | 0.00 |  | 0.00 | ± | 0.00 |  | 58.9 |
| 9 | 0.00 | ± | 0.00 |  | 0.00 | ± | 0.00 |  | 48.0 |
| 12 | 0.07 | ± | 0.01 |  | 0.03 | ± | 0.00 |  | 54.1 |
| 15 | 0.10 | ± | 0.02 |  | 0.06 | ± | 0.02 |  | 44.2 |
| 18 | 0.22 | ± | 0.07 |  | 0.10 | ± | 0.02 |  | 55.4 |
| 21 | 1.24 | ± | 0.47 |  | 0.26 | ± | 0.08 |  | 78.6 |
| 24 | 1.63 | ± | 0.56 |  | 0.44 | ± | 0.02 |  | 73.1 |
| 27 | 2.44 | ± | 0.62 |  | 0.70 | ± | 0.15 |  | 71.3 |
| 30 | 2.26 | ± | 0.19 |  | 0.47 | ± | 0.03 |  | 79.3 |
| 33 | 2.53 | ± | 0.28 |  | 0.67 | ± | 0.05 |  | 73.4 |
| 36 | 2.64 | ± | 0.36 |  | 0.69 | ± | 0.04 |  | 73.7 |
