# Supplementary material for: Lactobacillus delbrueckii ssp. bulgaricus OLL1073R-1-derived exopolysaccharide suppresses murine norovirus replication by modulating innate immune response
Source: PLoS One. 2026 Sep 2;21(9):e0356886. doi: 10.1371/journal.pone.0356886 (PMC13537558; doi:10.1371/journal.pone.0356886)
Supplement: Supplementary file 2 [file pone.0356886.s008.docx]

**S1 Protocol**

**Cell proliferation assay**

Cell viability of RAW264.7 cells were measured using Premix WST-1 Cell Proliferation Assay System (Takara Bio, Shiga, Japan) according to the manufacturer’s protocol. Briefly, RAW264.7 cells were seeded in a 12-well tissue culture plate and incubated for 24 h. The cells were incubated in growth medium (untreated control) or medium containing 200 µg/mL R-1 EPS for 12 or 36 h. After treatment, 10% premix WST-1 reagent was added to the culture medium and the absorbances at 450 nm and 690 nm were measured after 4 h incubation.

**Virucidal assay**

To assess whether R-1 EPS possesses direct virucidal activity against murine norovirus (MNV), a virucidal assay was performed. MNV was incubated either in the presence or absence of 200 µg/mL of R-1 EPS at 37 °C for 24 h. Following incubation, samples were subjected to ultracentrifugation at 186,000 g for 90 min to separate viral particles from unbound R-1 EPS. The recovered virus fraction was resuspended and adjusted to a multiplicity of infection (MOI) of 1, and incubated for 2 h at 4°C with RAW264.7 cells for infection. After incubation, the cells were washed with phosphate buffered saline once. The infected cells were incubated in growth medium for 24, and 48 h.

**Quantification of cytokines**

The levels of IFN-α and IFN-β in the culture supernatant of MNV-uninfected RAW264.7 cells with R-1 EPS treatment were measured. RAW264.7 cells were seeded in a 12-well tissue culture plate and incubated for 24 h. The cells were incubated in growth medium (untreated control) or medium containing 200 µg/mL R-1 EPS for 12, 24, and 36 h. After incubation, the culture supernatant was collected and the concentrations of IFN-α and IFN-β in the culture supernatant were determined using the VeriKine Mouse Interferon alpha ELISA Kit (PBL Assay Science, Piscataway, NJ, USA) and Quantikine ELISA Mouse IFN-β Kit (R&D Systems, Minneapolis, MN, USA), respectively, according to the manufacturers’ protocol. According to each protocol, absorbance at 450 nm was measured for the quantification of IFN-α, while absorbance at both 450 nm and 570 nm was measured for the quantification of IFN-β.

The concentrations of interleukin (IL)-1α, IL-1β, IL-6, IL-12, tumor necrosis factor (TNF)-α, macrophage inflammatory protein (MIP)-1α, MIP-1β, and keratinocyte chemoattractant (KC) were determined using the Bio-Plex Pro Mouse Cytokine Group1 Custom Panel (Bio-Rad, Hercules, CA, USA).
